# Major Bioactive Compounds from Java Plum Seeds: An Investigation of Its Extraction Procedures and Clinical Effects

**DOI:** 10.3390/plants12061214

**Published:** 2023-03-07

**Authors:** Gitishree Das, Rajat Nath, Anupam Das Talukdar, Duygu Ağagündüz, Birsen Yilmaz, Raffaele Capasso, Han-Seung Shin, Jayanta Kumar Patra

**Affiliations:** 1 Research Institute of Integrative Life Sciences, Dongguk University-Seoul, Goyang-si 10326, Republic of Korea; 2 Department of Life Science and Bioinformatics, Assam University, Silchar 788011, Assam, India; 3 Department of Nutrition and Dietetics, Faculty of Health Sciences, Gazi University, Emek, Ankara 06490, Turkey; 4 Department of Biological Sciences, Tata Institute of Fundamental Research, Hyderabad 500046, Telangana, India; 5 Department of Agricultural Sciences, University of Naples Federico II, 80138 Naples, Italy; 6 Department of Food Science & Biotechnology, Dongguk University-Seoul, Goyang-si 10326, Republic of Korea

**Keywords:** java plum seeds, diabetes, clinical effects, bioactive compounds, jambosine, extraction, mode of action

## Abstract

Java plum is widely recognized as a plant with valuable medicinal properties, originating from Indonesia and India and distributed globally in the tropic and sub-tropic regions of the world. The plant is rich in alkaloids, flavonoids, phenyl propanoids, terpenes, tannins, and lipids. The phytoconstituents of the plant seeds possess various vital pharmacological activities and clinical effects including their antidiabetic potential. The bioactive phytoconstituents of Java plum seeds include jambosine, gallic acid, quercetin, β-sitosterol, ferulic acid, guaiacol, resorcinol, *p*-coumaric acid, corilagin, ellagic acid, catechin, epicatechin, tannic acid, 4,6 hexahydroxydiphenoyl glucose, 3,6-hexahydroxy diphenoylglucose, 1-galloylglucose, and 3-galloylglucose. Considering all the potential beneficial effects of the major bioactive compounds present in the Jamun seeds, in the current investigation, the specific clinical effects and the mechanism of action for the major bioactive compounds along with the extraction procedures are discussed.

## 1. Introduction

*Syzygium cumini* (L.) Skeels., more popularly referred to as black Jamun or Java plum, belongs to the family Myrtaceae, and is a vital indigenous plant with medicinal applications originally from India and Indonesia; it is distributed in the tropics and sub-tropics around the globe [1,2,3]. The plant is fast-growing and can grow 30 m or more in height and its lifespan is more than 100 years [4]. The plant is treated as economically important as all of its parts, starting from the seeds and leaves to the wood, have great medicinal and economical values [2,5]. The plant possesses various phytoconstituents and has high antioxidant potential, which is very much beneficial for our bodies. It possesses phytoconstituents that include glucoside, anthocyanin, steroids, phenols, flavonoids, and terpenoids [6]. The fruit is rich in carbohydrates, vitamins, and minerals; the fruit pulp contains some important minerals including manganese, calcium, potassium, iron, zinc, and sodium [7,8].

**Figure 1:**
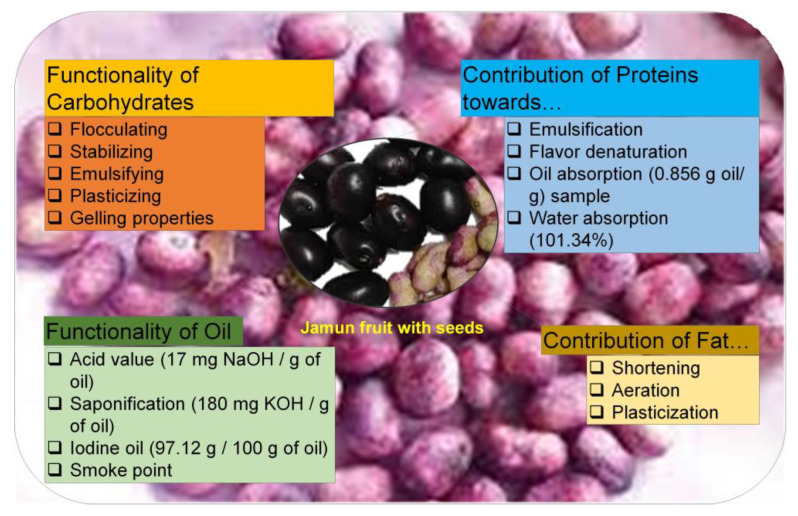
Functional properties of Jamun seeds. Adopted and reproduced from Kumar et al. [10], under the terms and conditions of the Creative Commons Attribution (CC BY) license (https://creativecommons.org/licenses/by/4.0/), 2022, Licensee MDPI, Basel, Switzerland (Originally Figure 1 in the main source).

Its purple-to-blackish color is the result of the anthocyanins present within the plant [9]. Other than the fruits, leaves and bark also have medicinal properties [2]. They are used in diabetes, ringworm, and diarrhea [1,2]. The bark is used as a digestive anthelmintic and diuretic [4]. In addition, seeds are used in various traditional and oriental systems of medicine such as in Ayurvedic, Unani, and Chinese medicines as a natural substitute for the treatment of hyperglycemia, ulcers, dysentery, asthma, glycosuria, and bronchitis [2,5]. The functional properties of the Jamun seeds as food are shown in Figure 1 [10].

**Figure 2:**
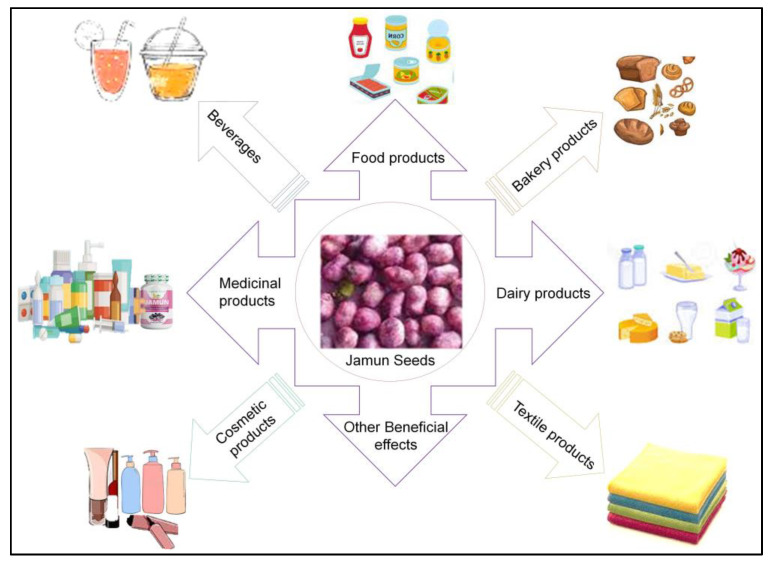
Multiple uses of Jamun seeds. Adopted and reproduced from Kumar et al. [10], under the terms and conditions of the Creative Commons Attribution (CC BY) license (https://creativecommons.org/licenses/by/4.0/) 2022, Licensee MDPI, Basel, Switzerland (Originally Figure 2 in the main source).

The seeds have promising high levels of alkaloids, jambosine, and glycoside jamboline or antimellin compounds known for stumbling starch’s diastatic conversion into sugars that makes it a good nutraceutical against type 2 diabetes [11,12,13]. As an overview, pharmacological activities of the plants include chemoprotective, analgesic, hypoglycemic, anti-inflammatory, anti-allergic, anti-oxidant, antihyperlipidemic, hyperglycemic, antiplaque, astringent to bowels, antimicrobial, gastro-protective, antidiarrheal, and antibacterial [2,6]. Moreover, there are reports that the functionally altered seed fibers are used for different applications in the food sector industries such as a thickener, bulking agent, a replacement of fat, and as dietary supplements. [10]. Some of these applications of Jamun seeds are shown in Figure 2 [10].

Considering all these potential properties of Jamun seeds, in this review, we exclusively describe the clinical effects and mode of action of the major bioactive constituents present in Jamun seeds.

## 2. Major Bioactive Constituents

Jamun seed, located in the center of the Jamun fruit, is 1–2 cm long and has a slightly bitter taste [14]. Jamun contains many phytochemicals, and not all the dominant bioactive constituents in different parts are the same (flower, fruit, seed, fruit pulp, leaves, and stem bark) [9]. The nutritional and phytochemical composition of Jamun fruit may differ depending on the maturity level, climatic conditions, growing region, agricultural practices, and post-harvest processing steps [15].

The nutritional composition of Jamun fruit appears to contain significant amounts of carbohydrates (including glucose, sucrose, fructose, and galactose), protein (asparagine, alanine, glutamine, tyrosine, and cysteine; free amino acids), vitamins (ascorbic acid, thiamine and niacin), and minerals (potassium, calcium, sodium, phosphorous, and iron) [16]. Jamun has furthermore been confirmed to possess various bioactive constituents that are shown to be supportive of human health [17]. The color, taste, and aroma of Jamun are closely related to the number of phytochemicals (polyphenols, tannins, and gallic acid) in its composition [9].

The nutritional composition of Jamun seed may differ slightly from its fruit. Raza et al. [18] analyzed the Jamun seeds’ and Jamun fruits’ nutritional composition and found the seeds to have the following approximate composition (%): moisture level of 16.34 ± 0.49, ash level of 2.18 ± 0.06, crude protein level of 1.97 ± 0.59, crude fat level of 0.65 ± 0.01, and crude fiber level of 4.19 ± 0.12; and calculated the composition of Jamun fruit as 82.19 ± 2.46, 2.04 ± 0.06, 2.15 ± 0.06, 0.83 ± 0.02, and 1.76 ± 0.05, respectively [18]. In another study, Jamun seed composition (%), in light of the results obtained from previous studies, has been reported as moisture 3.21–53, ash 1.5–21.72, carbohydrate 6.05–89.68, fiber 1.21–16.9, protein 1.97–8.5, fat 0.65–4.86, ascorbic acid 1.84–35.75, and iron (mg) 1.25–18.62 [19]. Jamun seeds were recently analyzed by collecting the fruits of ripe Jamun grown in Brazil, and the amount of moisture, ash, carbohydrate, protein, and lipid (%) was calculated as 62.25 ± 6.32, 0.36 ± 0.02, 14.95 ± 6.09, 19.96 ± 0.00, and 2.47 ± 0.24, respectively [20]. The amount of oil contained in Jamun seed varies according to its fruit, and although the solvent used is different, the dominant fatty acid is oleic acid [21,22]. The fatty acids present in Jamun seed oil were reported to include oleic acid-32.2%, myristic acid-31.7%, linoleic acid-16.1%, stearic acid-6.5%, palmitic acid-4.7%, vernolic acid-3.0%, lauric acid-2.8%, sterculic acid-1.8%, and malvalic acid-1.2% [23]. 

**Figure 3:**
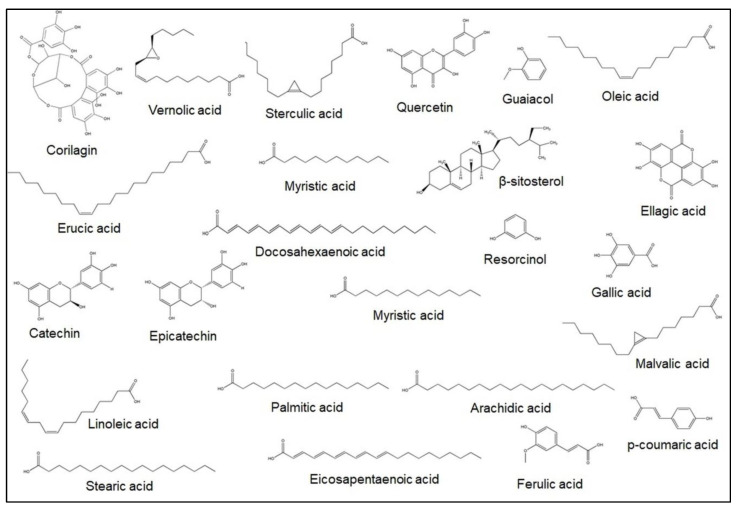
Primary bioactive constituents in Jamun seed.

More recently, fatty acid extraction of Jamun seed was carried out using different solvents. The fatty acid composition in this analysis using hexane is as follows (%); oleic acid-26.8, linoleic acid-25.2, palmitic acid-19.9, stearic acid-6.4, linoleic acid-2.6, arachidic acid-1.2, eicosapentaenoic acid-0.6, erucic acid-0.5, myristic acid-0.4, docosahexaenoic acid-0.3, and lauric acid-0.3 [21]. This plant species also contains significant amounts of many bioactive constituents including jambosine, quercetin, β-sitosterol, gallic acid, guaiacol, resorcinol, *p*-coumaric acid, corilagin, ellagic acid, catechin, epicatechin, and tannic acid [24,25]. Jamun’s total flavonoid contents (TFC), total phenolic contents (TPC), and antioxidant capacity differ among the assorted parts of the plant. Ali et al. [25] found that the amount of TPC (mg GAE/g), TFC (mg quercetin/100 g), anthocyanin (mg cyanidin 3-rutinoside equivalent/100 g), and free-radical scavenging capacity (%) in Jamun fruit skin is higher than in the pulp and seed [25]. The amount of the Jamun seed’s bioactive constituents may also differ depending on the type of solvent used [26]. Researchers furthermore found that methanol in the extraction of flavonoids is more effective than methylene chloride [27]. Bajpai et al. [28] determined that the antioxidant activity of the plant’s seeds was 85.49 ± 0.8% and total phenolic contents were 108.79 ± 1.0 mg/g gallic acid equivalent. Moreover, some of the major bioactive constituents of Jamun seed are illustrated in more detail in Figure 3 and Table 1.

## 3. Clinical Effects Imparted

**Figure 4:**
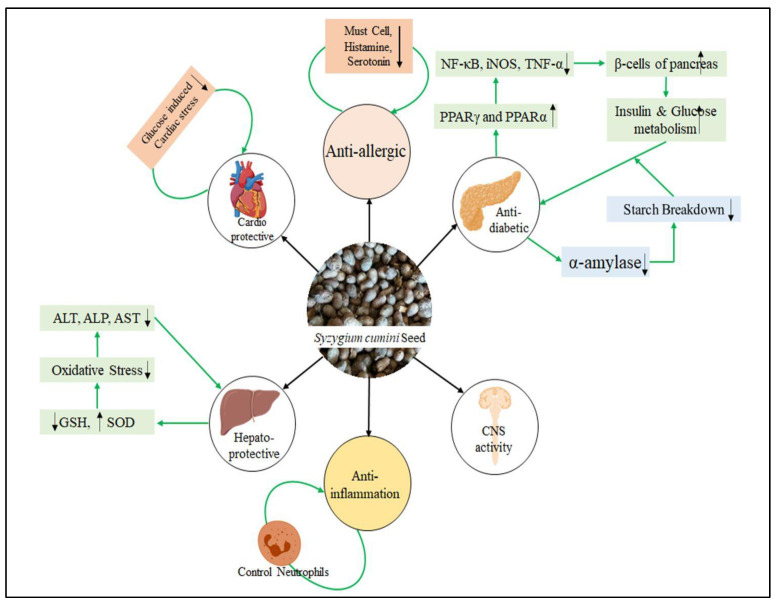
Pharmacological activity of Jamun seeds.

As mentioned before, the bioactive metabolites found in Jamun and the possible health effects from these metabolites may be different according to the considered parts of the Jamun fruit (pulp, leaf, seed, etc.), botanical type, and/or genotype, extraction techniques, and solvents used to obtain bioactive metabolites [12,13,32]. Although there are predominantly phenolic acids, flavonoids, and tannins, such as anthocyanins, flavonols, flavanols, and flavanonols, in the fruit, the amount of these varies according to the parts of the fruit [33]. Especially in the part of the seed, there are mainly phenolic compounds, including lignans, tannins, coumarins, gallic acid, stilbenoids, ferulic acid, and phloroglucinol derivatives, as well as some flavonoid compounds (e.g., quercetin, rutin, flavones, flavan-3-ols, flavonols, flavanonols, and dihydrochalcones) [1,13,34]. Examination of the evidence-based data in the literature shows that due to these bioactive compounds in its composition, Jamun seeds have anti-inflammatory, cardioprotective, hepatoprotective, anticancer, antimicrobial, and especially antioxidant effects and they can have a regulatory effect on blood glycemia through various mechanisms [33]. However, although it has often been pointed out that there are a limited number of clinical studies on this subject and the possible clinical effects of seeds’ extract forms were evaluated in both cell culture applications and laboratory animal models, the nutraceutical value of this pharmacological agent for many diseases makes it more and more popular day by day [32]. Various pharmacological activities of Jamun seeds are presented in Figure 4. Recently, do Nascimento-Silva et al. [2] summarized a variety of animal experiment studies related to the health-beneficial effect of Jamun seeds, which signifies how beneficial Jamun seeds are (Table 2). 

### 3.1. Phenolic Compounds

Phenolic acids may be best described as non-flavonoid phenolic substances that derive from benzoic acid and cinnamic acid and exist in the free or conjugated (soluble and insoluble) form [40]. They are largely present in plant species and have roles in a promisingly diverse assortment of cellular processes that have been shown to date to include growth and reproduction in plant physiology, and are secondary metabolites generated in the processes of defensive mechanisms in the fight against an array of environmental stressors [41]. Work conducted to date on phenolic acids shows that consuming them regularly has an effective therapeutic potential in protecting humans from various health problems [42]. The underlying mechanism here is that phenolics demonstrate antioxidant activities because they are scavengers of superoxide radical anions, hydroxyl radicals, some organic radicals, singlet oxygens, peroxyl radicals, and peroxynitrite, and, in addition, these compounds function as reducing agents and chain breakers [43]. Moreover, in the case of the phenolic acids, the gallic and the ellagic acids and their various simple and polymeric forms such as gallotannins and ellagitannins are studied enormously [24,34,44,45]. Balyan and Sarkar have found that around 21.9% and 8.65% of gallic acid and ellagic acid are present in the aqueous extract of seeds [24]. With the help of advanced techniques such as liquid chromatography–mass spectrometry (LC-MS) and nuclear magnetic resonance (NMR) spectroscopy, various researchers have identified around seventeen ellagitannins and nine gallotannins from the Jamun seeds [6,45,46].

Apart from these, there are other types of phenolic acids such as caffeic acids (1.02–4.73 µg/g seed), chlorogenic acids (0.89–6.80 µg/g seed), ferulic acids (1.50–8.21 µg/g seed), and *p*-cumaric acids (14.06 µg/g seed), that has been identified from the Jamun seeds [30,47]. Other authors have also identified 5-hydroxyveratric acid, syringic acid, and protocatechuic acid in the Jamun seeds [45,46]. Due to the high phenolic content such as ellagitannins and ellagic acid in Jamun seeds, it has been proven in many studies that it has high levels of antioxidant activities [29,48]. In research supporting this, Aqil et al. [49] performed the antioxidant analysis of the Jamun seeds using different methods (oxygen radical absorbance capacity (ORAC), 2,2-diphenyl-1-picrylhydrazyl (DPPH) assay, 2,2′-azino-bis(3-ethylbenzthiazoline-6-sulphonic acid) (ABTS) assay, and ferric reducing antioxidant power (FRAP) assay). Other authors have also proved that hydrolyzable tannins have strong antioxidant and antibacterial properties [50,51]. In another study, the author studied the antioxidant potential of Jamun seeds after the extraction of phenolic compounds [52]. The author concluded the potent antioxidant properties of the Jamun seeds in terms of the DPPH (IC_50_ value 0.24 μg/mL), ABTS (IC_50_ value 0.31 μg/mL, hydroxyl, metal chelating (IC_50_ value 48.39 μg/mL), nitric oxide radical scavenging (IC_50_ value 37.01 μg/mL), and lipid peroxidation inhibitory activity (IC_50_ value 5.38 μg/mL) [52]. In light of these studies, it can be said that the antioxidant potential of Jamun seeds brings this fruit to the forefront, especially in terms of its protective effect against oxidative stress-related diseases. 

It is determined that the major polyphenols such as ellagic, gallic, cinnamic, ferulic, and syringic acids present in *S. cumini* seeds can modulate and reduce tertiary butyl hydrogen peroxide (TBHP)-induced oxidative stress–angiotensin converting enzyme (ACE), β-Hydroxy β-methylglutaryl-CoA (HMG-CoA) reductase, and low-density lipoprotein (LDL) oxidation in H9c2 cardiomyoblasts and it may have a cardioprotective effect and this effect has been attributed to Jamun phenolics and flavonoids [53]. Other researchers successfully proved that *S. cumini* seed extract (0.9 mg/kg × 2 doses) has demonstrated protective effects to combat the systemic toxicity generated with methylmercury in neonatal rats and reduced the increased N-acetyl-β- kidney and urine and the level of lipid peroxidation observed in the kidney and liver, and also modulates adenosine deaminase (ADA) activities occurring within the hippocampus [54]. This protective effect is understood to be the outcome of antioxidant activities caused by phenolic acids including chlorogenic and gallic acids found in Jamun seed extract and rutin compounds [54]. It has been furthermore determined that both ethanolic acidic pulp and seed extract with high polyphenol content obtained from *S. cumini* have high oxygen radical absorbance (respectively; 1.445 ± 64 μmol Trolox equivalent (TE)/g vs. 3.379 ± 151 µM of TE/g) together with DPPH and ABTS scavenger and ferrous ion chelation abilities [29].

Similarly, in other research, it was successfully demonstrated that administering *S. cumini* seed extract for 5 days has a reducing effect on oxidative stress against strand breaks induced by hydroxyl radicals in pBR322 DNA and reduces genomic damages in an in vivo setting [26]. It was determined in a study that dietary intake of Jamun seed extract for 26 weeks delayed tumor development in breast cells of Swiss albino mice, decreased the incidence of tumors, and reduced tumor multiplicity and burden. Jamun has been reported to reduce breast cancer by significantly balancing the estrogen-mediated changes in breast cell proliferations, estrogen receptor-alpha (ER-α), cyclinD1, and candidate miRNAs (overexpression of miR-375 and miR-182 or underexpression of miR-206 and miR-127) [55]. It has been stated that many bioactive substances (including phenolics, oleanolic acid, betulinic acid, and dimethyl cardamonins) found in *Syzygium* spp. also have anticancer activity and this is often seen in conjunction with inhibited cell proliferation and the induction of apoptotic events [56]. It is reported that Jamun has an antiproliferative effect upon exposure to A549 cells, a human lung cancer line, and the major bioactive compounds that may have this effect originate from the ellagic acid/ellagitannin (0.5%) in seeds and anthocyanin (0.54%) and ellagic acid/ellagitannin (0.17%) in pulp powder [29]. As supported in this study, mainly ellagitanines, phenolic acids, and flavonols found in *S. cumini* seeds may have a chemoprotective effect [29]. Likewise, ellagitannins, especially the ellagic acid part, are known to exert strong therapeutic effects in many types of cancer, such as prostate, colon, breast, and skin [57]. Ellagic acid can have this effect through an assortment of diverse mechanisms, such as the inducing of pathways of apoptosis in many cell lines, preventing damage to DNA induced by carcinogens, and the inhibition of metastasis, along with various previously described anti-inflammatory and antioxidant mechanisms [57]. 

In addition, *S. cumini* was known throughout history to have antidiabetic potential employing its antihyperglycemic and hypoglycemic properties to different degrees, and has been used in alternative and complementary medicine before the discovery of insulin [58,59]. The effects of Jamun seed powder in glycemic control for individuals with type 2 diabetes were also researched in a randomized controlled study which determined fasting plasma glucose to decrease by 30.0%, post-prandial plasma to decrease by 22.0%, and hemoglobin A1c (HbA1c) to decrease from 8.99% to 8.31% in diabetic individuals with poor glycemic control after a 10 g/day seed powder supplementation applied for 90 days [60]. Additionally, the said antidiabetic effect may be the result of a single component or a specific combination of components including phenolic acids and flavonoids which may exert indirect or direct effects on insulin resistance and β-cell function of the seed extract of *Syzigium cumini* [60]. In another pilot study, 1 g of a combination of raw and cooked bitter gourd, Jamun seeds, and fenugreek seeds, was given to 60 non-insulin-dependent rats for 1.5 months, followed by 2 g, and after 3 months, blood sugar was improved in the diabetic rats and the need for oral hypoglycemic drug intake decreased [61]. There is even evidence suggesting that this effect is dose dependent (500–200 mg/kg body weight (BW) [62]. 

The dry seeds and their boiled extract have produced hypoglycemic effects and both the aqueous extract (5 g kg^−1^) and alcoholic extract (100 mg kg^−1^) of the seeds have shown positive effects against tissue damage in the brain of diabetic rats [63]. The potential mechanism often suggested by clinical studies evaluating the regulation of blood glucose by Jamun seed is that Jamun modulates carbohydrate metabolism and stimulates insulin secretions from the pancreas (β-cells) [64]. In a study that supports this, it was determined that both α-glucosidase and α-amylase were inhibited in vitro after gallic acid and catechin and *S. cumini* seed kernel extract with high bioactive substance content were combined, and this combination may have antidiabetic potential [65]. The ability to inhibit protein tyrosine phosphatase 1B and aldose reductase has been shown by the ethyl acetate fraction of seeds containing, amongst other valuable compounds, 5-furfural, valoneic acid dilactone, and rubuphenol (IC_50_ values: 0.77, 0.165, and 0.12 mg/mL, respectively); aldoreductase inhibition was determined. It has been determined that dilactone, rubuphenol, and ellagic acid have protein tyrosine phosphatase 1B activity (IC_50_: respectively; 9.37, 28.14, and 25.96 mg/mL) and that seeds have an antidiabetic effect because they contain these bioactive compounds [6]. 

Similarly, in one of the recent studies, a total of 21 phenolics from Jamun seeds that may have antidiabetic potential have been isolated and identified, and it was determined that they may demonstrate antidiabetic potential by reducing the levels of advanced glycation end products (AGEs) that are formed and inhibiting the activities of enzymes such as alpha-glucosidase that hydrolyze carbohydrates [45]. There are also some findings in the literature showing that extracts of different parts of Jamun reduce hepatotoxicity in rats. Especially, pulp aqueous, methanol, and other alcohol extracts are reported to decrease serum levels of liver enzymes, increase antioxidant enzyme levels such as glutathione peroxidase, decrease lipid peroxidation, and thus decrease fibrosis and necrosis [66,67,68]. These effects are thought to be caused by natural antioxidants such as gallic acid and anthocyanin [69]. Considering this information, the phytochemical contents and antioxidant activities of seeds and extracts obtained from Jamun can be promising for hepatoprotective activity, which should be taken into consideration in further clinical trials. In light of these studies, it can be said that the Jamun seed phenolics and Jamun seed extracts as a combination of bioactive compounds have an antioxidant effect and play a promising role in both preventing and treating many ailments that occur in association with oxidative states.

Flavonoids are secondary metabolites found in many fruits and vegetables as aglycones or glycosides. Chemically flavonoids have basic structures of 15-carbon skeletons that comprise one heterocyclic and two phenyl rings. Flavonols, flavones, flavonoids, flavanones, anthocyanidins, and isoflavones are important flavonoids [70]. It has been reported by several authors, that among the type of flavonoids found in the Jamun seeds, catechin, quercetin, kaempferol, and epicatechin are found abundantly [30,47]. Moreover, myricetin and its derivatives such as myricetin glycosides, syringetin, and laricitrin are widely present in the seeds [45,46]. While the main flavonoid found in the pulp of *S. cumini* is anthocyanins, there are other important phytochemical constituents in the seeds of Jamun such as quercetin and rutin, and myricetin [57]. Among these compounds, quercetin, kaempferol, and rutin are mainly responsible for their effective bioactive potentials [71]. It is to be noted that the anthocyanins which are found in the fruit pulp are absent in the seeds, which might be affected during the ripening stage of the fruit [4,29]. Some other authors have also detected resveratrol, coumarins, phloroglucinol derivatives, lignans, etc. in the Jamun seeds [30,72]. Additionally, resveratrol has been reported to be responsible for the high antioxidant potential of the Jamun seeds [30].

In one study, Gajera et al. [47] measured the levels of quercetin in seed (0.04 ± 0.001 µg/g) and kernel (0.05 ± 0.002 µg/g) parts of only one Jamun species in their analysis with six different Jamun species [47]. Quercetin, a flavonoid, is among the dietary antioxidants of the highest significance [73]. Recently, it has been emphasized that quercetin might have the capability of exerting helpful effects in both prophylaxis and treatment in cases of the novel coronavirus disease (COVID-19) due to its anti-inflammatory, strong scavenger, and thrombin-inhibitory properties [74,75]. Flavonoids such as quercetin are among the promising target compounds that will surely be explored in clinical trials against COVID-19 infections in the future due to their pleiotropic activities and the fact that they do not cause systemic toxicity [76]. Alongside its antiviral effects, studies over the years have shown that quercetin can be considered for applications in treating many disorders or diseases including type 2 diabetes mellitus, cardiovascular disease, osteoporosis, many different cancers, blood pressure, and mental-cognitive and pulmonary disorders [77]. Therefore, as a source of quercetin, the powder forms of the parts of Jamun such as seeds (total flavonoids: 457.2 ± 31.44 mg 100 g^−1^) and pulp (total flavonoids: 36.00 ± 5.95 100 g^−1^) are used to increase the antioxidant capacity of foods as well as to increase their clinical benefits [73,78]. It has a special ability to scavenge highly reactive species such as superoxide anions or hydrogen peroxide that may trigger oxidative damage within cellular components such as lipids, proteins, and DNA acids. In this way, it has important protective roles in pathophysiological and degenerative processes caused by oxygen radicals [79].

In particular, its anticancer activity comes to the fore and it can play therapeutic roles against many diseases encompassing malignancies, inflammatory disorders, and oxidative stress [80]. In this context, there are some hypotheses that quercetin can be used as an adjuvant or stand-alone treatment in many metabolic diseases [81]. In one study, 50 Sprague–Dawley rats (all male) with diabetes induced by the administration of streptozotocin were given 30 mg/kg of quercetin for 14 days; their elevated serum blood glucose and insulin decreased, together with their dyslipidemia, and oxidative stress and tissue injury biomarkers, and hyperlactatemia and ketoacidosis markers, which are complications of diabetes, all dramatically improving [81]. Quercetin, given at doses of 15 mg kg/day for four weeks, has also been found to decrease blood sugar and raise the level of plasma insulin in rats with diabetes induced by administering streptozotocin and simultaneously improved bone mineral metabolism and structural bone matrix [82]. There is also some evidence that quercetin may have positive effects on obesity and its comorbidities, which are associated with low-grade inflammation, due to its anti-inflammatory effects [83].

It has been suggested that the performance of quercetin is due to the result of the ability to alleviate intracellular oxidative stresses, reduce low-grade chronic inflammation, inhibit lipogenesis and adipogenesis, and suppress preadipocytes differentiating from matured adipocytes [84]. In one study, it was reported that quercetin has an antiobesity effect via the signaling pathways of adenine monophosphate-activated protein kinase and mitogen-activated protein kinase [85]. In addition, it has also been found in a study conducted on Wistar rats that it has a corrective effect on dysbiosis within the intestinal microbiota caused by diets that are high in fats as a result of administering a mixture containing quercetin at 30 mg/kg body weight and resveratrol at 15 mg/kg body weight daily for 10 weeks [86]. In addition, in a systematic meta-analysis study reviewing the effects of quercetin on levels of blood pressure, it was reported that quercetin supplementation at doses of >500 mg/day may have a significant effect in reducing blood pressure [87]. Similarly, in another study, it was found that quercetin may have an anti-hypertensive effect by activation of Na⁺-K⁺-2Cl^−^ cotransporter 1 (NKCC1) within the epithelial cells of the kidneys [88]. In light of these clinical studies, it is obvious that Jamun seed may have important clinical benefits as a source of quercetin, but more randomized controlled clinical studies are needed in this context.

Another important flavonoid found in Jamun seeds with clinical implications is rutin. Rutin, a type of flavonol, is found in many plants. In clinical terms, it has been reported that it exerts various biological effects, increasing the strength of blood capillaries and performing antihypertensive, antioxidant, and alpha-glucosidase inhibitory activities [89]. A study by Khan et al. [90] showed that the ethyl acetate fraction of *S. cumini* seeds displayed encouraging antimutagenic and DNA-protective activity, and its flavonoid constituents, including rutin, contributed ominously to the experiential activity. 

Jamun, which has a complex matrix of both flavonoid content and other phytochemicals, can scavenge free radicals, increasing the antioxidant capacity of cells, and reducing lipid peroxidation as a result of the increased levels of major antioxidant enzymes. Furthermore, it can suppress transcription of nuclear factor kappa B, inducible nitric oxide synthase, peroxisome proliferator-activated receptor, tumor necrosis factor-alpha, and many other pro-inflammatory cytokines, simultaneously modulating the upregulating of the transcription of nuclear factor erythroid 2-related factor 2, and it has important physiological roles in the antioxidant system and the associated clinical problems [91]. 

In addition to its antioxidant effects, compounds such as 3,5,7,4′-tetrahydroxy flavanone found in the seeds of this plant were also identified as amylase inhibitors [92]. Similarly, in another study, it has been determined that the flavonoids and other functional groups in the seed can inhibit the alpha-amylase enzyme by up to 96%, depending on the dose, and thus have an antidiabetic effect [93]. Chemically, rutin is a compound with low water solubility (0.125 g/L), which makes it difficult to integrate into functional foods and nutritional supplements [94]. This solubility-related disadvantage highlights Jamun as a potential functional nutrient because of its potential clinical benefits and as a source of rutin.

Tannins are another important secondary plant metabolite. Although they have different classifications according to their chemical structures and stability, they are generally classified as hydrolyzable, complex, and/or proanthocyanidins [95]. In Syzygium cumini fruit, there are some hydrolyzed and condensed tannins. Hydrolyzed tannins, defined as ellagitannins, consist of glucose in the center and gallic and ellagic acid units surrounding it. As condensed tannins, there are B-type oligomers of epiafzelechin (propelargonidin) with different degrees of polymerization [96]. However, in one study conducted, it was determined in an HPLC-DAD-ESI-MS/MS analyses that non-tannin phenolics are mostly predominant in the skin part of the Jambolan fruit in *S. cumini* and the existing tannin varieties are hydrolyzable tannins (equal amounts of gallotannins and ellagitanins) [97]. In addition, it has been reported that extraction using acetone can extract tannins better than ethanolic extraction [98]. 

In the literature, it has been stated that the tannins extracted from the fruit and seeds of this plant have strong DPPH radical scavenging and ferric-reducing/antioxidant activities, and therefore, this fruit is a natural antioxidant source [96]. Some tannins come to the forefront with antiviral, antibacterial, and antitumor activities [99]. Studies with *S. cumini* seed tannins are mostly related to their gastroprotective and antidiabetic effects. One study determined that *S. cumini* tannins (20.0 g tannins/kg) reduce gastric damage by reducing free-radical formation in the stomach in Sprague–Dawley rats with induced gastric mucosal damage and thus have an antiulcerogenic effect [100]. In another interesting study, it was reported that ellagic acid and urolithin A (3,8-dihydroxy-6H-dibenzopyran-6-one), which are colonic metabolites of ellagitannins (total phenolic substance: 20.5% GAE), which are hydrolyzable tannins found in Jamun, reduce colon carcinogenesis in human 293T cell line by modulating Wnt pathway-mediated transcriptional activation, and especially, urolithin A is a bioactive metabolite that is effective in modulating gut microbiota [101].

In a preclinical assessment of diabetes, 250, 500, and 1000 mg/kg doses of powdered seeds (identified bioactive components: tannins, gallic acid, oxalic acid, and triterpenoids) were supplemented to STZ-induced diabetic rats for 15 days, and especially, the seed powder at 500 and 1000 mg/kg doses caused antidiabetic effects on fasting blood sugar, peak blood sugar, and liver glycogen stores. In the same study, a subacute toxicity phase 2 study was also conducted and it was demonstrated that the 2.5 and 5.0 g/kg seed powder did not cause deaths or any morbidity [102]. In another study, three novel hydrolyzable tannins, namely iso-oenothein C and jamutannins A and B, were identified in Jamun seeds, in addition to ellagitannin, which has an antidiabetic effect similar to acarbose, which is one of the oral antidiabetic drugs, and it was reported that they have α-glucosidase inhibitory effects [103].

### 3.2. Terpenes and Terpenoids

In *S. cumini*, there are monoterpenoids (e.g., linalool oxide, 1,8-cineole, nerol, terpinolene, citronellol) and triterpenoids (e.g., acetyl oleanolic acid, oleanolic acid), and sesquiterpenes at a lower level [104]. It has demonstrated that these compounds, also known as the essential oil fraction of *S. cumini* seeds, have anti-inflammatory, antimicrobial, antihyperlipidemic, and chemopreventive effects [105,106]. Researchers assessed the anti-inflammatory effects (total leukocyte, neutrophil, and eosinophil migration) of *S. cumini* seed essential oil (100 mg/kg) in male Swiss mice by a lipopolysaccharide-induced pleurisy model and it was determined that eosinophil migration decreased by 67%. It was determined that this inhibitory effect was correlated primarily with β-caryophyllene and β-pinene found in the oil, and α-pinene increased this effect synergistically [105]. 

Betulinic acid, which has recently come to the fore among terpenes, is a bioactive compound also known as pentacyclic lupine-type triterpenoid and is isolated from different parts of Jamun [107,108]. In the studies on betulinic acid and its derivatives, it has been often reported that it has therapeutic and anti-HIV and antimalarial activities against anti-inflammatory, antidiabetic, antihyperlipidemic, and nonalcoholic fatty liver disease and it has even been reported that it has an antiviral effect against Dengue virus (DENV) due to its antioxidant effect [108,109,110,111]. It has been even stated that it is an important antineoplastic/chemopreventive agent, which has been discovered recently [112]. A study determined that betulunic acid causes apoptosis of differentiated PC12 cells caused by ROS via the mitochondrial apoptotic pathway [106]. In an experimental study that supports this, it was reported that betulinic acid (50 μM) can induce apoptotic processes in ovarian A2780 cells via mitochondria-dependent and -independent pathways and may be considered a novel chemopreventive agent [113]. It has been determined by other researchers that betulinic acid inhibits pancreatic cancer formation through mTOR-caspases/Bcl2/Bax apoptotic signaling modulation and may have a chemopreventive effect [114]. It has even been reported that it is safe in mice up to 500 mg/kg body weight [109]. Considering the results of these studies, it is predicted that Jamun, which is a valuable provider of both betulinic acid and assorted bioactive substances, will become more popular day by day and the number of clinical studies to be conducted in this context will increase.

### 3.3. Alkaloids

Alkaloids are nitrogenous organic molecules and naturally occurring [115]. Several researchers have reported the presence of alkaloids and their beneficial effects in the Jamun seeds [116,117]. Hasanuzzaman et al. reported on the presence of alkaloids in the acetone and chloroform seed extracts of the *S. cumini* fruit [118]. They also stated that alkaloids are used as medicines in serious illnesses including cancer, heart failure, and blood pressure, besides their applications as euphoric and addicting drugs and pesticides or insect repellents [118,119,120]. Older reports have shown the anti-inflammatory effects and antioxidant properties of the seed extract on rats [119,121,122]. Moreover, some studies have also proved the beneficial effects of seeds on diabetic humans [119,120,123]. Other older research on the seeds has shown that the seed extracts have a high-hypoglycemic effect on diabetic rabbits [124]. Ayurveda’s traditional system of medicine has suggested that the average dose of 1–3 g of seed powder per day is ideal [125]. Moreover, there are no side effects of seed and bark extracts in the traditional reports, but high-tannin bark extracts might source slight gastrointestinal distress in some people if taken with food [120]. Some authors have written that the Jamun seeds contain an alkaloid, jambosine, which helps in the halting of the diastatic conversion of starch into sugar [116,126]. The seed extract has lowered blood pressure by 34.6% and this act is attributed to the ellagic acid content in the seeds [126]. Rajkumar et al. reported finding alkaloid contents of 81.07 mg/g in the seed extract [115]. Alkaloids are highly useful in the management of diabetes and they are responsible for the reverse conversion of starch into sugar in human blood levels [115]. Despite the useful and medicinal properties of Jamun, some adverse effects of Jamun have also been reported in humans, such as lowering blood sugar [91]. Hence, it was suggested to avoid its consumption after immediate surgery or by pregnant women, etc. [91].

## 4. Major Extraction Procedures

Many factors such as the extraction methods of Jamun seeds, solvents used in extraction, and duration of extraction cause significant changes in extracted bioactive constituents and reaction kinetics [24]. This naturally leads to the potential health benefits, which are medicinal properties, attributed to Jamun seeds [29,127]. The most common and important methods used to extract the major bioactive components of Jamun seed and the results of these methods are given in Table 3. While Jamun seed contains about 3–10% oil, the main fatty acids are reported as myristic acid and oleic acid [128]. Soxhlet extraction (SJE) and gas chromatography–mass spectrometry (GC-MS) are the main methods in Jamun seed oil analysis. While the Soxhlet method is a traditional technique used to extract fat from foods, GC-MS is a prominent method in the analysis of fatty acids. Although the Soxhlet method has some disadvantages, it is one of the most popularly applied techniques due to its unattended and simple use [129]. While the oil content of Jamun seed was reported as 10% in one of the two different studies using the Soxhlet method, it was calculated as 2.47% in the other study [20,128]. Another reliable and frequently used method for the quantitative extraction of lipids and the extraction of fatty acids is the Folch method [130]. Although it is frequently used in the oil analysis of some plant seeds, no study has been found in the literature in which Jamun seed has been analyzed by this method.

The extraction of phenolic compounds in fruits such as Jamun is recommended from fresh samples. However, due to the high perishability of these fruits, methods such as freeze-drying are generally used. The phenolic contents may be seriously affected by the particular approaches used to prepare the extract [49]. While certain methods used in the preparation of seed extracts from this plant provide high efficiency for phenolics, on the other hand, some methods in the literature may have problems with reproducibility [33].

Different methods are used in the analysis of the bioactive components of Jamun seed. While the most common traditional methods are the conventional solvent extraction method and SJE, modern methods include UJE or MJE, ultrafiltration, solid-phase microextraction, and supercritical fluid extraction [117]. do Carmo Brito et al. [136] emphasized that ethanol 95% with 1% of HCl (*v*/*v*) is the most efficient extraction method to extract anthocyanins from Jamun fruits [136]. In another study, as a result of analysis using different extraction methods, the total phenolic contents (mg GAE/100 g), flavonoid content (mg quercetin equivalents (QE)/100 g), and total anthocyanin contents (mg cyanidin-3-glucoside equivalent (CYE)/g) were obtained with the highest amount in the ethanol extracts of both Jamun fruit and seeds [131]. This was followed by methanol and water extracts [131]. In addition to the extraction technique and the characteristics of the solvent used, pH is also one of the important factors in the analysis of bioactive components; especially anthocyanins [136]. Although different results are obtained in different analysis methods, an ideal method for the extraction of the bioactive components of Jamun seed has not been reported yet.

## 5. Conclusions and Future Perspectives

Jamun is easily available, and highly nutritious, with multiple medicinal properties. Along with its taste, the fruit is loaded with flavonoids, phenyl propanoids, alkaloids, tannins, terpenes, and lipids. The phytoconstituents of Jamun seeds have pharmacological activities such as antidiabetic, hepatoprotective, antiallergic, and cardioprotective properties which support its facts, i.e., Jamun is a highly health-beneficial fruit. It also discloses numerous medicinally imperative bioactive compounds existing in Jamun seeds and validates their use as a conventional medication for the management of different diseases. Among all the pharmacological aspects, Jamun seed is widely popular for its antidiabetic activity. As diabetes is a disease of concern globally, Jamun might assume a crucial future role in controlling hyperglycemia by ingesting it in the raw form. Moreover, it is highly significant to study the phytoconstituents of Jamun seeds and explore their possibilities in finding a cure for diabetes or other related diseases.

## Figures and Tables

**Table 1:** The major bioactive constituents of Jamun seed.

Sample Characteristics	Collected From	Main Bioactive Constituent	Reference
Dry Jamun seed powder	Maharashtra, India	**Phenolic compounds (mg/g DE)** Tannic acid: 188.5 ± 5.6 Gallic acid: 90.8 ± 2.7 Ellagic acid: 36.0 ± 1.1 Caffeic acid: 26.07 ± 0.8 Catechin: 9.05 ± 0.27 Quercetin: 1.54 ± 0.04 Epicatechin: 0.42 ± 0.01 *p*-coumaric acid: 0.26 ± 0.03 TPC (mg GAE/g DE): 415 ± 10 TFC (mg/g DE): 44.1 ± 1.4	[24]
Jambolana fruits, in the mature stage	Viçosa, Minas Gerais, Brazil	TPC (mg GAE/g): 22.59 ± 0.79	[20]
Jamun seed extracts	National Institute of Pharmaceutical Education and Research’s gardens, S.A.S. Nagar, India	TPC (g/GAE): 0.07–0.12. Total anthocyanins (g): not detected Soluble sugar (g): 0.001. Free ellagic acid (mg): 36.34–44.25.	[29]
Jamun seed coat (fresh weight basis) ^a^ and ethanol extract of the seed coat (dry weight) ^b^	Three locations within Thiruvananthapuram, Kerala province, south India	Total free phenol (g/kg): 8.1 ± 0.8 ^a^ and 270.0 ± 3.4 ^b^. Flavonoids (g/kg): 0.41 ± 0.08 ^a^ and 25.30 ± 0.37 ^b^.	[4]
Fresh Jamun seeds, 4 different extracts (ethanol, aqueous, ethyl acetate, acetone)	Tiruchirappalli, India	**TPC contents (mg GAE/g)** Ethanol: 471.67 ± 29.3 Aqueous: 168.33 ± 7.64 Ethyl acetate: 375 ± 40.93 Acetone: 230 ± 25 **TFC contents (mg quercetin/g)** Ethanol: 114.88 ± 5.36 Aqueous: 65.31 ± 1.77 Ethyl acetate: 138.26 ± 6.58 Acetone: 103.86 ± 3.67 **Antioxidant activity (IC_50_ values, µg/mL)** Aqueous: 25.02 Ethanol: 24.53 Acetone: 24.29 Ethyl acetate: 24.42	[26]
Jaman fruit parts	Potowar region of Pakistan	TPC (mg GAE/g): 4812.03 ± 10.67 TFC (mg quercetin/100 g): 2380 ± 5.08 Anthocyanin (mg cyanidin 3-rutinoside equivalent/100 g): 272.26 ± 6.04	[25]
Jamun seed ethanol/water extract (80:20 *v/v*)	Central Sericulture Research and Training Institute, Central Silk Board, Mysore, India	TPC (mg GAE/g dry weight): 55.54 ± 2.06 TFC (mg CE/g dry weight): 5.09 ± 0.28 DPPH content (IC_50_ values) 0.40 ± 0.00 mg/mL TAC: 3.33 ± 0.10 mM GAE g^−1^ of extract	[30]
Jamun seed extracts	Different natural habitats, India	Phenolic substances (µg/g plant material, dry weight) Gallic acid: 646 Quercetin: 98 Kaempferol: 59 Ellagic acid: 38 Caffeic acid, ferulic acid, and rutin: not shown AOA: 85.49 ± 0.8% TPC: 108.79 ± 1.0 mg/g	[28]
Ripe/matured Jamun fruit	Obtained in Manikganj district, Bangladesh	Tannins, total (mg TAE/g dry extract): 617.85 ± 5.32 **Phenolic compounds (100 mg/g dry extract)** Gallic acid: 70.59 ± 0.77 Catechin hydrate: 28.11 ± 0.13 Vanillic acid: 17.01 ± 0.10 Cafeic acid: 16.27 ± 0.08 Syringic acid: 5.39 ± 0.05 Epicatechin: 48.32 ± 0.54 Vanillin: 8.85 ± 0.07 Trans-ferulic acid: 1.07 ± 0.02	[31]

DE: dry extract; TPC: total polyphenol contents; TFC: total flavonoid contents; GAE: gallic acid equivalents; TAC: total antioxidant capacity; AOA: antioxidant activity; CE: catechin equivalents.

**Table 2:** A summary of various experimental studies on the health-beneficial effects of Jamun seeds in an animal model.

Biological Properties	Animal Model	Experimental Design	References
*Antidiabetic effect:* low blood glucose concentrations were observed in both treated groups after 60 days. Better results were observed in the seed-treated rats. The diet affected insulin levels momentously.	Sprague–Dawley rats.	Normal: 60 days; 3 groups (*n* = 5): (1) control; (2) 3% of lyophilized seed powder; (3) 3% of lyophilized pulp powder. Hyperglycemic: 60 days; 3 groups (*n* = 5): (1) control + 40% sucrose; (2) 3% of lyophilized seed powder + 40% sucrose; (3) 3% of lyophilized pulp powder + 40% sucrose.	[35]
*Antidiabetic effect:* glucose levels were reduced significantly and serum insulin levels also improved. Hypolipidemic effect: a reduction in total cholesterol, LDL-c, and serum triglyceride, and increased serum HDL-c were observed. *Hepatoprotective activity:* the extract treatment did not show any statistically significant reduction in serum creatinine and serum urea. However, the protein concentration increased significantly, even more than that of the rats treated with the gliclazide, and a reduction in the ALT, AST, ALP, and bilirubin levels was observed. A liver section revealed normal hepatic tissue.	Wistar albino rats of either sex (230–250 g), 8–10 weeks of age with diabetes (Alloxan: 150 mg/kg).	14 days; 5 groups (*n* = 6): (1) control—only vehicle; (2) diabetic control—only vehicle; (3) diabetic + methanolic extract of *S. cumini* seeds (100 mg/kg, p.o.); (4) diabetic + methanolic extract of *S. cumini* seeds (200 mg/kg, p.o.); (5) diabetic + gliclazide (25 mg/kg, p.o.).	[36]
*Antidiabetic effect:* there was a significant reduction in serum glucose, insulin, and HOMA-IR. The supplementation had a protective effect on β-cells of diabetic rats, evidenced by an increased architecture of nuclei and mitochondria. The effect was dose dependent. *Hypolipidemic effect:* the levels of total cholesterol, triglyceride, and LDL-c were reduced significantly, and an increased level of HDL-c was observed. *Effect on antioxidant activity:* the levels of superoxide dismutase, catalase, and glutathione peroxidase increased. The contents of thiobarbituric acid reactive substances and TNF-α decreased.	Male Wistar albino rats (150–200 g) 12–14 weeks old (STZ: 40 mg/kg)	21 days; 5 groups (*n* = 10): (1) control—saline (5.0 mL/kg, p.o.); (2) positive control—diabetic rats + saline; (3) diabetic + *S. cumini* (100 mg/kg; 200 mg/kg; 400 mg/kg); (4) diabetic + *Aegle marmelos* (500 mg/kg) + *S. cumini* (200 mg/kg); (5) diabetic + Metformin (100 mg/kg).	[37]
*Antidiabetic effect:* there was a significant decrease in blood glucose and HbA1c levels, and an increase in serum insulin levels and hemoglobin, toward normal levels. *Hypolipidemic effect*: the blood levels of total cholesterol, triglyceride, and LDL-c were reduced considerably, and an increase in the levels of HDL-c was observed. *Effect on antioxidant activity*: the levels of superoxide dismutase, catalase, glutathione peroxide, reduced glutathione, and thiobarbituric acid reactive substances were reversed significantly to near normal *Hepatoprotective activity*: the serum glutamic oxaloacetic transaminase and serum glutamic pyruvic transaminase significantly decreased.	Male Wister albino rats (150–200 g), 2–3 months of age	4 weeks; 4 groups (*n* = 6): (1) control—citrate buffer (0.1, pH 4.5; i.p); (2) positive control—STZ-induced diabetic rats. (3) STZ-induced diabetic rats + 200 mg *S. cumini*/kg b.w. orally, once weekly. (4) STZ-induced diabetic rats + 200 mg *C. zeylanicum*/kg b.w. orally, once weekly.	[38]
Antiamnesic activity: improved cognitive deficit, decreased the time required for a successful labyrinth test, ameliorated memory impairment improves the response to oxidative stress, inhibits acetylcholinesterase activity. Thus, it is comparable to the effect of piracetam. *Dose dependency*: better results with higher dosage.	Male albino Wistar rats (150–200 g), 6–8 weeks of age.	8 days; 5 groups (*n* = 6): (1) control—only vehicle; (2) positive control—only vehicle + scopolamine (1 mg/kg, i.p.)—induced amnesia; (3) standard drug—piracetam (200 mg/kg, i.p.) + scopolamine; (4) methanolic extract of *S. cumini* (200 mg/kg, p.o) + scopolamine; (5) methanolic extract of *S. cumini* (400 mg/kg, p.o) + scopolamine.	[39]

Results are expressed as mean ± SD. Reproduced with permission from do Nascimento-Silva et al. [2], 2022, Licensed Content Publisher: Elsevier (Originally, part of Table 5 from the main source).

**Table 3:** Some research results on the extraction of Jamun seeds’ major constituents.

Extraction Technique	Extraction Solvent	Extraction Duration or Range	Reported Optimum Extraction Conditions	Major Bioactive Compounds Obtained in Extraction	Reported Amount and Activity of Major Bioactive Compounds	Reference
Conventional solvent extraction method	Two different binary solvents methanol and ethanol with one aqueous phase	30–60 min	Using ethanolic extract Duration: 45 min	Flavonoids (+) Anthocyanin (−)	The maximum TPC: 1863.25 ± 70.83 mg GAE/100 g. The maximum flavonoids: 953.91 mg/100 g.	[131]
SJE	Petroleum ether	30 min	ND	Seed oil	Oil content: 10.0%	[132]
SJE	Hexane	ND	ND	Oleic acid n-hexadecanoic acid Cyclooctasiloxane, hexadecamethyl 1-monolinoleoylglycerol trimethylsilylether Octadecanal, 2-bromo- Cyclooctasiloxane, dodecamethyl- Cyclooctasiloxane, tetradecamethyl- Pyrazole [4,5-b] imidazole, 1-formyl-3-ethyl-6-á-d-ribofuranosyl- Stearic acid, 3-(octadecyloxy) propyl ester Benzaldehyde, 2,4,5-trimethox	Oleic acid: 30.28% n-hexadecanoic acid: 20.30% Cyclooctasiloxane, hexadecamethyl: 0.79% 1-monolinoleoylglycerol trimethylsilyl ether:1.45% Octadecanal, 2-bromo-:2.61% Cyclooctasiloxane, dodecamethyl-:0.79% Cyclooctasiloxane, tetradecamethyl-:0.69% Pyrazole [4,5-b] imidazole, 1-formyl-3-ethyl-6-á-d- ribofuranosyl-:1.63% Stearic acid, 3-(octadecyloxy) propyl ester:1.49% Benzaldehyde, 2,4,5-trimethox:39.98%	[133]
Different extraction methods- SJE, UJE, MJE	The ethanolic extracts for SJE, UJE, MJE	SJE: 10–12 cycles UJE: 40 s at 20 KHz MJE: at 100 °C for 50 s.	Extraction yields (%) SJE 9.83 ± 0.35 UJE: 12.76 ± 1.45 MJE: 11.65 ± 1.06	Saponins (+ for SJE) Alkaloids (+ for SJE, UJE, MJE) Phenols (+ for SJE, UJE, MJE) Tannins (+ for SJE, UJE, MJE) Flavonoids (+ for SJE, UJE, MJE) Glycosides (− for SJE, UJE, MJE) Carbohydrates (− for SJE, UJE, MJE) Phytosterols (− for SJE, UJE, MJE) Proteins (− for SJE, UJE, MJE)	TPC values (mg of GAE/100 mL) SJE:376.28 ± 6.11 84.73 ± 1.4 MJE: 399.39 ± 2.75 94.03 ± 1.6 UJE: 425.90 ± 15.2	[117]
Ultrafiltration (UF)	Distilled water with UF/ NF membranes	89.4 min	UF/NF methods can be used in extraction and storage, as well.	Original seed extract: tannic acid (most abundant) UF clarified extract: gallic acid (most abundant) NF concentrated extract: gallic acid (most abundant) NF permeated extract: gallic acid (most abundant)	TPC values: Original seed extract: 1560 ± 46 mg/L UF clarified extract: 910 ± 27.3 mg/L NF concentrated extract: 1266 ± 38 mg/L NF permeated extract: 300 ± 9 mg/L Most Abundant bioactives Original seed extract: tannic acid (692 ± 28 mg/L) UF clarified extract: gallic acid (282.5 ± 8.5 mg/L) NF concentrated extract: gallic acid (286.2 ± 8.6 mg/L) NF permeated extract: gallic acid (272.16 ± 8.16 mg/L)	[134]
MJE	ethanol	1–5 min + 30 min centrifuge	515.45 w of microwave power, 3.23 of pH, 3.15 min, 1:15.06 g/mL of solid-to-liquid ratio	Polysaccharide	Polysaccharide (4.71 ± 0.02%)	[135]

Footnote: ND: not defined; SJE: Soxhlet extraction, UJE: ultrasonication-assisted extraction, MJE: microwave-assisted extraction; GAE: gallic acid equivalent; UF/NF: ultrafiltration/nanofiltration; TPC: total polyphenol contents.

## Data Availability

All data related to this manuscript are available in the form of figures and tables in the manuscript.
